# Supplementary material for: Comparative genomics provides new insights into the diversity, physiology, and sexuality of the only industrially exploited tremellomycete: Phaffia rhodozyma
Source: BMC Genomics. 2016 Nov 9;17:901. doi: 10.1186/s12864-016-3244-7 (PMC5103461; doi:10.1186/s12864-016-3244-7)
Supplement: Additional file 6: — List of orphan genes with links to PFAM (related to Additional file 1: Table S1). (ZIP 1428 kb) [file 12864_2016_3244_MOESM6_ESM.zip › PFAM_UNIQUE/G01128_P.html]

#                                                                            --- full sequence --- -------------- this domain -------------   hmm coord   ali coord   env coord
  
# target name        accession   tlen query name           accession   qlen   E-value  score  bias   #  of  c-Evalue  i-Evalue  score  bias  from    to  from    to  from    to  acc description of target
  
#------------------- ---------- ----- -------------------- ---------- ----- --------- ------ ----- --- --- --------- --------- ------ ----- ----- ----- ----- ----- ----- ----- ---- ---------------------
  
G01128\_P             -            119 EspB                 PF05802.6    317     0.063   10.9   1.4   1   1   5.6e-05     0.067   10.8   1.4   118   153    31    67    17    95 0.74 - Enterobacterial EspB protein  
G01128\_P             -            119 HCV\_NS5a\_C           PF12941.2    244     0.013   13.7   0.8   1   1   5.3e-06     0.016   13.5   0.8   179   226    34    81    17    89 0.87 - HCV NS5a protein C-terminal region  
G01128\_P             -            119 RNA\_pol\_I\_A49        PF06870.7    385     0.098    9.9   1.1   1   1   4.1e-05      0.12    9.5   1.1   124   171    28    76    22    82 0.84 - A49-like RNA polymerase I associated factor  
G01128\_P             -            119 Tape\_meas\_lam\_C      PF09718.5     78      0.35    9.5   4.5   1   2       1.2   2.4e+03   -2.8   0.0    55    61     2     8     2    10 0.83 - Lambda phage tail tape-measure protein (Tape\_meas\_lam\_C)  
G01128\_P             -            119 Tape\_meas\_lam\_C      PF09718.5     78      0.35    9.5   4.5   2   2   6.6e-05      0.13   10.9   1.9    11    41    31    61    28    70 0.85 - Lambda phage tail tape-measure protein (Tape\_meas\_lam\_C)
